# Chronic Kidney Disease and Its Relationship with Mental Health: Allostatic Load Perspective for Integrated Care

**DOI:** 10.3390/jpm11121367

**Published:** 2021-12-14

**Authors:** Federica Guerra, Dina Di Giacomo, Jessica Ranieri, Marilena Tunno, Luca Piscitani, Claudio Ferri

**Affiliations:** 1 Department Mesva, University of L’Aquila, 67100 L’Aquila, AQ, Italy; federica.guerra@graduate.univaq.it (F.G.); dina.digiacomo@univaq.it (D.D.G.); claudio.ferri@univaq.it (C.F.); 2 Dyalisis Division, San Salvatore Hospital of L’Aquila, 67100 L’Aquila, AQ, Italy; mtunno@asl1abruzzo.it (M.T.); lucpis90@virgilio.it (L.P.); 3 Internal Medicine and Nephrology Division, San Salvatore Hospital of L’Aquila, 67100 L’Aquila, AQ, Italy

**Keywords:** end-stage renal disease, allostatic load, biomarkers, psychological dimensions, creatinine, psychological distress, stress, depression, anxiety

## Abstract

Background: Chronic renal failure is a chronic medical condition characterized by a progressive and irreversible loss of kidney function. Up to 50% of patients undergoing dialysis experience symptoms of depression and anxiety: what is the impact of individual factors and medical conditions on the mental health issue? The present study was carried out to investigate the individual factors (biomarkers and psychological dimensions) of end-stage renal disease patients dealing with dialysis, analyzing their predictor values for developing negative disease adaptations by an allostatic paradigm. Methods: We conducted an observational study on 35 patients affected by end-stage renal disease; biological and psychological markers have been detected. We conducted descriptive statistical analyses (*t*-tests) and performed a hierarchical regression analysis to investigate the relationship between pathological medical conditions and psychological dimensions. Results: The findings showed a positive correlation between creatinine levels and psychological distress as well as stress index. No significant effect of “time of dialysis”, “time from diagnosis”, “age” and “personality traits” was found. Conclusion: Our findings showed that personality traits did not represent a protective factor by moderating positive emotional adaptations; conversely, creatinine levels appeared predictive for negative emotional adaptations. High levels of creatinine were found to be positively associated with high stress levels as well psychological distress. According to the allostatic paradigm, end-stage renal disease patients could experience an allostatic load and more overload towards poor health outcomes; integrated biological and psychological measurements could prevent increased negative mental health through a patient-centered approach.

## 1. Introduction

The prevalence of chronic kidney disease (CKD) and resultant end-stage renal disease (ESRD) worldwide is nearly 11%, and it is the cause of 2.4 million deaths [1]. Chronic renal failure is a progressive and irreversible destruction of renal function.

Psychological distress, depression, and anxiety negatively impact the quality of life (QoL) and well-being of patients. Depression has been well documented as a common extra-renal comorbidity in approximately 30–40% of ESRD patients and is associated with increased mortality risk. Body image damage as well physical, functional, metabolic, social and mental fragility can affect the QoL of patients [2].

Hemodialysis (HD) is the common form of dialysis therapy for ESRD, and it is associated with a high burden of comorbidity and complications of ESRD due to the intrusiveness of treatment that is required: patients need to adapt themselves to eating and drinking restrictions, as well as fluid intake and chronic pain [3,4].

HD therapy is a stressful process, and its burden affects the daily living of patients, as regular therapy in the hospital and recovering from therapy impacts physical functioning and leads to negative emotions related to the progression of the disease and the onset of depression and anxiety [3]. The literature shows that 63.9% of HD patients showed anxiety, 60.5% demonstrated depression, and 51.7% reported stress; the prevalence of psychiatric hospitalization among HD patients is 1.5–3 times higher than other chronic diseases [4]. A total of 10% of HD patients demonstrated a history of psychiatric disorders, as compared to 2.5% in the general population. These disorders included recurrent suicide ideation, sexual disorders, interpersonal problems, paranoia, physical complaints, compulsive disorders, psychoses, aggression, and phobias [5].

When dealing with the side effects of HD, patients implement maladaptive coping strategies, and the consequence is decreased QoL [6,7]. Several studies have investigated the QoL pre- and post-kidney transplantation, showing high anxiety and depression levels in HD patients [8,9,10].

Lately, clinical practice and research interests have been focused on the interaction between individual and external aspects as factors affecting living and compromising the QoL in CKD therapy; personality dimensions proved to be predictive for adaptive behaviors [10]. Part of posttransplant nonadherence seems to be determined by personality. Patients with low conscientiousness may be criticized for their carelessness, negligence, and failure to stay within the lines, while patients with high conscientiousness are disciplined, organized, goal-oriented, and have a high need for structure, i.e., all characteristics that may help people in treatment adherence [11].

A study conducts by Thomas C.V. et al., 2016, which investigated the association between personality traits, clinical/biochemical changes (hypertension, infection, acute rejection, graft loss, and death) over a period of nine months after kidney transplant, showed that there was no difference in demographics and clinical/biochemical variables. In relation to the average levels of Estimated Glomerular Filtration Rate (eGFR) no significant effects were noted over time. However, personality dynamics have been shown to contribute to the mechanism of adaptation to the lived situation, allowing, according to the characteristics of each, greater or lesser capacity for autonomy and well-being [9].

Research indicates that agreeableness is related to health promotion behaviors and adaptation to dialysis [12,13].

According to the literature, CKD is a complex clinical condition related to mental health comorbidities. As reported above, several studies have focused on the mental health issues by detecting psychological negative aspects and emotional dimensions. However, few studies have analyzed mental health management in HD patients by pointing out the allostatic factors to identify how adaptive changes are related to dialysis therapy, referring to the process of allostatic load.

Allostatic state refers to “achieving stability through change” [14], and it is the active process that leads to adaptation to a stressor. Mediators of allostasis include stress hormones as well as the autonomic nervous system, pro-inflammatory cytokines and metabolic hormones. Allostatic load and its more severe form, allostatic overload, represent the cumulative effects of chronic physiologic stress, which may be generated by internal processes (e.g., anxiety) and by external factors such as chronic stressors or by lifestyles (e.g., overeating, insufficient sleep) that also dysregulate the mediators of allostasis. Consequences of allostatic overload include mental health disorders [15].

Based on clinical practice, we wanted to detect and analyze the internal factors of ESRD patients in regular and prolonged pharmacological treatments (dialysis) modulating the mental health adaptation to the chronic disease. Following that, the present study aimed to investigate the individual factors (biomarkers and psychological dimensions) of ESRD patients dealing with dialysis by analyzing their predictor values for developing negative disease adaptations. In particular, the current study aimed to analyze the dynamics of emotional dimensions in ESRD patients undergoing HD therapy to investigate mental health adaptations to HD therapy in chronic ESRD.

## 2. Materials and Methods

### 2.1. Participants

**Figure 1:**
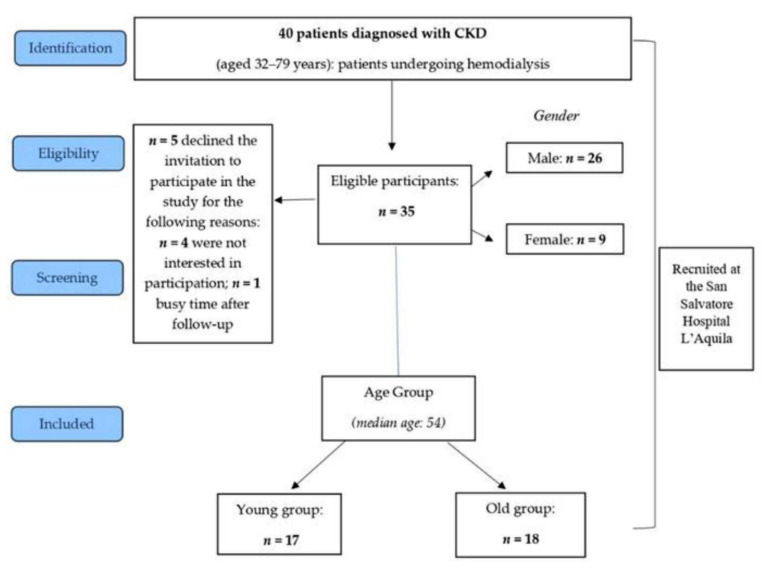
Flowchart of participants.

A total of 35 patients age range of 32–79 years (mean = 55.4; SD ± 11.3) affected by kidney disease participated in the study; the gender distribution of the sample reflects chronic renal failure epidemiology, as the disease is more prevalent in males (F = 9, mean age 54.8, SD ± 11.9; M = 26 mean age 55.5; SD ± 11.3). Education distribution was as follows: high school graduate (45.71%), graduate (7.15%), and not graduated (47.14%). Further, 58.57% of study participants were unemployed. Patients have been enrolled in S. Salvatore Hospital (L’Aquila, IT) for clinical follow-up and hemodialysis treatment. We contacted 40 eligible patients, and 35 of them provided informed consent. Demographic characteristics of the participants were reported in Table 1; the recruitment process was described in Figure 1.

Medical staff identified suitable patients, and recruitment was voluntary. Eligible participants met the following inclusion criteria: (a) CKD diagnosis; (b) undergoing hemodialysis therapy; (c) age > 18 years; (d) willingness to participate in the study and provide signed informed consent. The exclusion criteria were as follows: presence of serious chronic illnesses or significant physical or psychological disabilities that could invalidate informed consent or their responses.

### 2.2. Procedure

The medical staff of the San Salvatore Hospital identified suitable patients, who were then enrolled during clinical follow-up according to medical protocol during dialysis therapy. Informed consent was obtained at the time of enrolment. Trained clinical psychologists (blinded as to the study’s objectives) conducted the psychological assessments in a private, dedicated room. The evaluations lasted 20 min. The Dialysis Treatment participants (DT) completed the measurements during the dialysis treatment. The data was collected anonymously.

### 2.3. Measures

#### 2.3.1. Sociodemographic Variables

Demographic data were collected by self-reports detecting individual data (e.g., age, educational level, occupational status, having children, being employed, and marital status) and clinical data (diagnosis, dialysis therapy, dialysis timing, surgical intervention).

#### 2.3.2. Psychological Measurement

The psychological battery was composed of three standardized tests measuring emotional traits (depression, anxiety, stress, and psychological distress) and personality dimensions. Tests were applied following the clinical interview session. Each standardized test was applied using its Italian population adaptation.

Depression Anxiety Stress Scales 21 (DASS-21) [16]. The DASS-21 is a self-report that measures the degree of severity of the core symptoms for emotional dimensions rather than a categorical conception of a psychological disorder. It is composed of 21 questions with responses on a 4-point Likert-type scale, and it measures 3 sets of self-report scales designed to measure the emotional states of depression, anxiety, and stress. The inventory demonstrated good reliability (α = 0.90).

Psychological Distress Inventory (PDI) [17]. This self-administered questionnaire measures the impact of psychological distress and related therapies. It is composed of 13 questions, and responses are indicated on a 5-point Likert-type scale. The standard score estimates the presence/absence of psychological distress to measure global distress. This test was administered only to the participating group. The inventory demonstrated good reliability (α = 0.86).

Big Five Inventory-10 Italian Version (BFI-10) [18]. This self-administered questionnaire measures the five personality dimensions (agreeableness, conscientiousness, emotional stability, extroversion, and openness). It is composed of 10 questions with responses on a 5-point Likert-type scale. Agreeableness describes an individual’s tendency to put the needs of others before their own; conscientiousness describes a person’s tendency to be persistent and determined in achieving their goals; emotional stability describes an individual’s response to stress; extroversion refers to the degree of pleasure experienced through social relationships; openness refers to openness to creativity, non-conformism, and originality. The reliability of test was Cronbach’s a c > 90.

People with a high level of agreeableness are nice, cooperative, and accommodating; on the contrary, a low score in this domain is typical in competitive and self-interested people. People with a high level of conscientiousness tend to work hard to carry out their plans, while people who attain a low score on this trait tend to change course and get distracted easily. People with poor emotional stability are susceptible to anxiety, depression, anger, and other negative emotions when subjected to stressful conditions, but on the contrary, people with a high level of emotional stability resist stress and tend not to experience many negative emotions. A high score in extroversion indicates a person who is sociable, talkative, open to others, and optimistic; on the contrary, a low score is typical in reserved, sober, not euphoric, and quiet people. Low scores of openness indicate closure to experience, conformity, and lack of creativity, while high scores indicate ideas which are creative, original, and innovative.

### 2.4. Study Design

We conducted an observational study to evaluate the correlation between emotional traits and personality dimensions in ESRD patients. The data were carefully checked for possible coding errors or misattribution of values, and missing data were updated before the analysis was conducted. Participants were divided into groups based on gender (male, female) and age group (young, old by median value).

### 2.5. Statistical Analysis

Descriptive statistics (*t*-test) were conducted to analyze the characteristics of the sample, and hierarchical regression analysis was performed to investigate the relationship between pathological medical conditions and psychological dimensions.

The Jamovi stat was applied for statistical analyses. The level of significance adopted was α < 0.05.

## 3. Results

### 3.1. Correlation among the Study Variables

Table 2 reported the raw scores (mean values and standard deviations) obtained on the psychological testing battery.

One sample *t*-Test analysis showed a significant effect for the investigated emotional dimensions, as reported in Table 3.

Subsequently, we conducted a Spearman correlation analysis between emotional dimensions and creatinine levels, as reported in Table 4. Creatinine level is positively associated with PDI score and Stress index. Furthermore, PDI score is positively associated with depression (*r* = 0.78, *p* = 0.0001), anxiety (*r* = 0.55, *p* = 0.0006) and stress (*r* = 0.80, *p* = 0.0001), and finally, even anxiety and stress are associated with one another (*r* = 0.78 to 0.79, *p* = 0.0001). Because the variable “time of dialysis”, “time from diagnosis” and “age” are not significantly associated with any dependent variable, they were omitted from the subsequent analysis.

### 3.2. Testing Direct Effects and Moderating Effects

Considering the significant correlations between creatinine levels and emotional dimensions (stress and distress labels) in ESRD patients, a regression analysis was conducted to analyze the predictive effect, as reported in Table 5.

**Figure 2:**
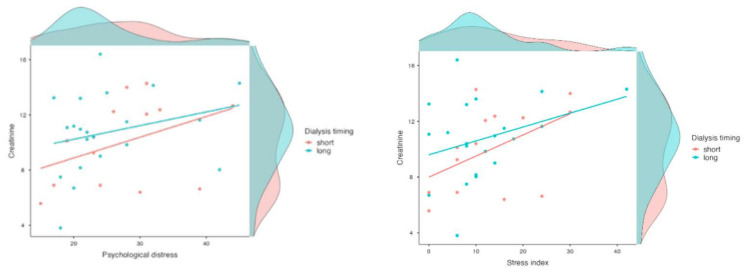
Scatter plots of negative emotions in dialysis groups divided by (median value) timing of treatment.

We included creatinine level and dialysis timing such that patients were divided into 2 groups by dialysis timing, taking the median value (median value = 24 months): T0 (HD therapy > 24 months) and T1 (HD therapy < 24 months). The main moderation model turned out to be significant (model 1: creatinine; model 2: dialysis timing). In model 1 (R^2^ = 0.14, F(1, 33) = 5.56, *p* = 0.02), it was revealed that the stress index was moderated by creatinine level and not dialysis timing. In model 2 (R^2^ = 0.16, F(1, 33) = 3.11, *p* = 0.05), a borderline effect emerged (Figure 2).

No significant correlational effect between emotional dimensions and personality traits was found.

Subsequently, Spearman’s correlation was conducted to investigate the relationship between emotional dimensions and personality traits. Data elaboration evidenced no significant effect among them.

## 4. Discussion and Conclusions

The aim of the current study was to investigate the relevance of the allostatic load of ESRD patients regarding HD therapy. Biomarkers and psychological dimensions were analyzed to understand their predictive value.

As expected, the findings of the present study demonstrated the impact of HD therapy in this chronic condition, confirming negative emotional signs such as anxiety, depression, stress, and psychological distress [4,5,6,7,8,9,10]; several researchers highlighted that HD patients were reported to have fewer functional coping strategies and had more mental health issues. Some authors highlighted the impact of personality traits on health management when dealing with high/low adherence and compliance to medical prescriptions [9,10,11,12,13]: positive traits can enhance the active care from the patients being engaged in their own health behavior.

According to the literature, our study highlighted the psychological unbalancing of HD patients, but offered a new perspective joining the (positive and/or negative) psychological dimensions to their medical records. Furthermore, and as expected, our findings showed that personality traits can be the individual factors driving enhanced physical caring for mental health. However, our findings showed the involvement of personality traits in health management, but they can be considered a protective factor for HD patients, as no moderating effect emerged for positive emotional adaptations. That is, ESRD patients seemed to suffer negative emotions related to biomarker levels and personality traits, and psychological dimensions did not help them to deal significantly with the impact of the side-effects of chronic disease. Conversely, creatinine levels (biomarkers) appeared predictive for negative emotional adaptations: high level of creatinine were found to be positively associated with high stress levels as well as psychological distress.

Our findings suggested that the managing of ESRD patients’ mental health is featured by changing the time dependently to the variability of biological markers (in our case creatinine) impacting psychological dimensions. According to the allostatic paradigm [16], ESRD patients could experience an allostatic load and more overload towards poor health outcomes; integrated biological and psychological measurements could prevent negative mental health-related outcomes. The complex measurement process could be even more decisive in the detection of mental health comorbidities, allowing for the tailoring of psychological interventions to decrease the negative impact of toxic stress on health.

One interesting point within this study was the absence of a significant correlation among personality traits and emotional dimensions: positive personality traits did not seem to be adequate protective factors when dealing with the impact of HD treatments over time. In our opinion, this finding is evidence for the need for the health management of ESRD patients by a multidisciplinary team by a holistic approach based on physical and psychological well-being interventions. By adopting the above operative perspective based on allostatic load in ESRD, the power of integrated assessment could contribute to a better understanding of the signs and symptoms planning the care. The patient-centered approach, in which the mental and physical interaction is central, is enhanced by the allostatic paradigm towards the improvement of the quality of life of ESRD patients in HD treatment.

There are some limitations to this study. This is a single-center study with a small sample. Regardless, the power of our finding is enough to be relevant for clinical practice because it is in line with a previous study [9]. The impact of personality traits could be positive on the medical pathway and also contribute to adaptations in the clinical experience, allowing different degrees of wellbeing according to the reduced influence of personality dynamics on health management. Furthermore, the patients in this study were enrolled in a non-random way, which would result in bias which could interfere with the evaluation of effectiveness. Therefore, a large sample size and multi-center randomized controlled trial is needed in the future. Lastly, another limitation of the study was the period detection data: as reported above, the detection data was in the range time from January 2020 to June 2021 during the COVID-19 worldwide pandemic. However, we monitored psychological stress for COVID-19 and all patients included in the sample showed no related emotional issue.

## Figures and Tables

**Table 1:** Demographic characteristics of the sample.

	DT (*n* = 35)
Age (years)	X55.4 SD ± 11.32
Age groups: *n* (%)	
≤54 years	17 (48.57)
>54 years	18 (51.43)
Gender: *n* (%)	
Male	26 (74.3)
Female	9 (25.7)
Marital status: *n* (%)	
Single	9 (25.7)
Married	26 (74.3)
Educational level: *n* (%)	
High school graduate	18 (51.43)
No graduate	17 (48.57)
Occupational status: *n* (%)	
Unemployed	21 (60.00)
Employed	14 (40.00)

Note: DT = dialysis treatment; SD = standard deviation.

**Table 2:** Raw score (mean/standard deviation) by psychological evaluations distributing sample in patient condition groups.

	DT	Total
	x SD	x SD
PDI	26.1 ± 7.9	25.7 ± 9.4
DASS-21		
Depression	9.03 ± 9.4	8.43 ± 10.2
Anxiety	8.46 ± 6.3	9.31 ± 8.3
Stress	12.2 ± 9.6	12.0 ± 9.6
BFI-10		
Ag	6.51 ± 1.99	6.26 ± 1.9
Co	7.66 ± 2.1	7.41 ± 2.1
Es	5.69 ± 1.4	5.73 ± 1.6
Ex	6.49 ± 2.1	6.44 ± 2.0
Op	6.83 ± 2.2	6.71 ± 2.1

Note: DT (dialysis treatment); SD (Standard Deviation); PDI (Psychological Distress Inventory); DASS-21 (Depression, Anxiety, Stress Scale); BFI-10 (Big Five-10); Ag (agreeableness); Co (conscientiousness); Es (emotional stability); Ex (extroversion); Op (openness).

**Table 3:** One sample *t*-test analysis on emotional dimensions of ESRD patients.

		Statistic	df	*p*	Mean Difference		Effect Size
PDI index	Student’s t	19.30	34.00	<0.0001	26.06	Cohen’s d	3.26
Depression	Student’s t	5.63	34.00	<0.0001	9.03	Cohen’s d	0.95
Anxiety	Student’s t	7.84	34.00	<0.0001	8.46	Cohen’s d	1.33
Stress	Student’s t	7.46	34.00	<0.0001	12.17	Cohen’s d	1.26

Note: df (degree of freedom); PDI (Psychological Distress Inventory).

**Table 4:** Correlation matrix (Spearman test) comparing emotional dimensions and creatinine levels.

		Creatinine	Distress	Depression	Anxiety	Stress	Time of Dialysis	Age	Time from Diagnosis
Creatinine	Spearman’s rho								
	*p*-value								
Distress	Spearman’s rho	0.39							
	*p*-value	0.0199							
Depression	Spearman’s rho	0.20	0.78						
	*p*-value	0.2441	<0.0001						
Anxiety	Spearman’s rho	0.01	0.55	0.78					
	*p*-value	0.9619	0.0006	<0.0001					
Stress	Spearman’s rho	0.36	0.80	0.75	0.63				
	*p*-value	0.0352	<0.0001	<0.0001	<0.0001				
Time of Dialysis	Spearman’s rho	−0.05	−0.19	−0.02	−0.07	−0.15			
	*p*-value	0.7965	0.2761	0.8937	0.6756	0.4005			
Age	Spearman’s rho	−0.14	−0.05	0.05	0.09	0.13	0.25		
	*p*-value	0.4209	0.7569	0.7712	0.6250	0.4637	0.1472		
Time from diagnosis	Spearman’s rho	0.09	0.09	0.17	−0.01	0.05	0.38	−0.11	
	*p*-value	0.5957	0.5876	0.3162	0.9735	0.7912	0.0253	0.5187	

**Table 5:** Regression analysis on stress and dialysis timing covaried by creatinine level.

Overall Model Test						
Model	R^2^	Adjusted R^2^	F	df1	df2	*p*
1	0.14	0.12	5.59	1	33	0.0241
2	0.16	0.11	3.11	2	33	0.0584

Note: R^2^ (coefficient of determination); F (Fisher); df (degree of freedom).

## Data Availability

The data presented in this study are available on request from the corresponding author.
